# Detection of subjects and brain regions related to Alzheimer's disease using 3D MRI scans based on eigenbrain and machine learning

**DOI:** 10.3389/fncom.2015.00066

**Published:** 2015-06-02

**Authors:** Yudong Zhang, Zhengchao Dong, Preetha Phillips, Shuihua Wang, Genlin Ji, Jiquan Yang, Ti-Fei Yuan

**Affiliations:** ^1^School of Computer Science and Technology, Nanjing Normal UniversityNanjing, China; ^2^Division of Translational Imaging and MRI Unit, New York State Psychiatric Institute, Columbia UniversityNew York, NY, USA; ^3^School of Natural Sciences and Mathematics, Shepherd UniversityShepherdstown, WV, USA; ^4^School of Electronic Science and Engineering, Nanjing UniversityNanjing, China; ^5^Jiangsu Key Laboratory of 3D Printing Equipment and ManufacturingNanjing, China; ^6^School of Psychology, Nanjing Normal UniversityNanjing, China

**Keywords:** Alzheimer's disease, Welch's *t*-test, magnetic resonance imaging, machine learning, machine vision, eigenbrain, support vector machine, particle swarm optimization

## Abstract

**Purpose:** Early diagnosis or detection of Alzheimer's disease (AD) from the normal elder control (NC) is very important. However, the computer-aided diagnosis (CAD) was not widely used, and the classification performance did not reach the standard of practical use. We proposed a novel CAD system for MR brain images based on eigenbrains and machine learning with two goals: accurate detection of both AD subjects and AD-related brain regions.

**Method:** First, we used maximum inter-class variance (ICV) to select key slices from 3D volumetric data. Second, we generated an eigenbrain set for each subject. Third, the most important eigenbrain (MIE) was obtained by Welch's *t*-test (WTT). Finally, kernel support-vector-machines with different kernels that were trained by particle swarm optimization, were used to make an accurate prediction of AD subjects. Coefficients of MIE with values higher than 0.98 quantile were highlighted to obtain the discriminant regions that distinguish AD from NC.

**Results:** The experiments showed that the proposed method can predict AD subjects with a competitive performance with existing methods, especially the accuracy of the polynomial kernel (92.36 ± 0.94) was better than the linear kernel of 91.47 ± 1.02 and the radial basis function (RBF) kernel of 86.71 ± 1.93. The proposed eigenbrain-based CAD system detected 30 AD-related brain regions (Anterior Cingulate, Caudate Nucleus, Cerebellum, Cingulate Gyrus, Claustrum, Inferior Frontal Gyrus, Inferior Parietal Lobule, Insula, Lateral Ventricle, Lentiform Nucleus, Lingual Gyrus, Medial Frontal Gyrus, Middle Frontal Gyrus, Middle Occipital Gyrus, Middle Temporal Gyrus, Paracentral Lobule, Parahippocampal Gyrus, Postcentral Gyrus, Posterial Cingulate, Precentral Gyrus, Precuneus, Subcallosal Gyrus, Sub-Gyral, Superior Frontal Gyrus, Superior Parietal Lobule, Superior Temporal Gyrus, Supramarginal Gyrus, Thalamus, Transverse Temporal Gyrus, and Uncus). The results were coherent with existing literatures.

**Conclusion:** The eigenbrain method was effective in AD subject prediction and discriminant brain-region detection in MRI scanning.

## Introduction

Alzheimer's disease (AD) is not a normal part of aging. It is a type of dementia that causes problems with memory, thinking, and behavior. Symptoms usually develop slowly and worsen over time. Symptoms may become severe enough to interfere with daily life, and lead to death (Hahn et al., 2013). There is no cure for this disease. In 2006, 26.6 million people worldwide suffered from this disease. AD is predicted to affect 1 in 85 people globally by 2050, and at least 43% of prevalent cases need high level of care (Brookmeyer et al., 2007). As the world is evolving into an aging society, the burdens and impacts caused by AD on families and the society has also increased significantly. In the US, healthcare on people with AD currently costs roughly $100 billion per year and is predicted to cost $1 trillion per year by 2050 (Miller et al., 2012).

Early and accurate detection of AD is beneficial for the management of the disease (Han et al., 2011). Presently, a multitude of neurologists and medical researchers have been dedicating considerable time and energy toward this goal, and promising results have been continually springing up (Xinyun et al., 2011). Magnetic resonance imaging (MRI) is an imaging technique that produces high quality images of the anatomical structures of the human body, especially in the brain, and provides rich information for clinical diagnosis and biomedical research (Shamonin et al., 2014). The diagnostic values of MRI are greatly enhanced by the automated and accurate classification of the MR images (Goh et al., 2014; Zhang et al., 2015a,b). It already plays an important role in detecting AD subjects from normal elder controls (NC) (Angelini et al., 2012; Smal et al., 2012; Nambakhsh et al., 2013; Hamy et al., 2014; Jeurissen et al., 2014).

In earlier cases, most diagnosis work was done to measure manually or semi-manually a priori region of interest (ROI) of magnetic resonance (MR) images, based on the fact that AD patients suffer more cerebral atrophy compared to NCs (Kubota et al., 2006; Anagnostopoulos et al., 2013). Most of these ROI-based analyses focused on the shrinkage of hippocampus and cortex, and enlarged ventricles (Pennanen et al., 2004). Somehow, the ROI-based methods suffer from some limitations. First, the methods focus on the ROIs need prior knowledge. Second, the accuracy of early detection depends heavily on the experiences of the examiners. Third, the mutual information among the voxels is difficult to operate (Xinyun et al., 2011; Lee et al., 2013). Finally, there is no evidence that other regions (except hippocampus and entorhinal cortex) did not provide any information related to AD. Also, the auto-segmentation of ROI is not feasible in practice, and examiners tend to segment the brain manually.

On the other hand, multivariate approaches that consider all the voxels in a scan as one observation offer an alternative method to ROI-based methods. The advantages of multivariate approaches are that they are data driven, which means that the analyses are fully based on the data without any prior knowledge and that the interactions among voxels and error effects are assessed statistically. However, multivariate approaches suffer from either the curse of dimension problem or the small sample size problems or the lack of the capability, to make statistical inferences about regionally specific changes (Álvarez et al., 2009b).

The Eigenbrain was an excellent multivariate approach that solves both the curse of dimensionality and the problems in small sample size. It was proposed by Alvarez et al. (2009a) and Lopez et al. (2009), and was applied on Single Photon Emission Computed Tomography (SPECT) images. In their research, the eigenbrain approach was shown to efficiently reduce the feature space from ~5 × 10^5^ to only ~10^2^, and therefore, was able to achieve excellent classification accuracy. In this study, we make a tentative test of applying eigenbrains in MRI scans for AD detection.

Support vector machine (SVM) has been arguably regarded as one of the most excellent classification methods in machine learning (Zhang and Wu, 2012a). Original SVMs are linear classifiers, and do not perform well on nonlinear data. Hence, we introduced in the kernel SVMs (KSVMs), which extends original linear SVMs to nonlinear SVM classifiers by applying the kernel function to replace the dot product form in the original SVMs (Gomes et al., 2012). Compared with the original plain SVM, the KSVMs allows one to fit the maximum-margin hyperplane in a transformed feature space (Garcia et al., 2010). The transformation may be nonlinear and the transformed space is high dimensional; thus although the classifier is a hyperplane in the high-dimensional feature space, it may be nonlinear in the original input space (Hable, 2012).

The aim of our study was to develop a novel classification system based on eigenbrain and machine learning, in order to grow a computer-aided diagnosis (CAD) system for the early detection of AD subjects and AD-related brain regions. Our goal was not to replace clinicians, but to provide an assisting tool. The rest of the paper was organized as follows: the next section reviewed relates literatures from two aspects: the extracted features and the classification methods. Section The Proposed Method describes the methodology of the proposed CAD. Section Experiments and Results contains the experiments and results. Section Discussion analyzes the reason behind the experiment results. Finally, Section Conclusion and Future Research is devoted to conclusion and future research. For ease in reading, the acronyms and their meanings of this study are listed in Table 12 in the appendix.

The **contributions** of the paper fell within the following five aspects: (i) We generalized the Eigenbrain to MR images, and proved its effectiveness; (ii) We proposed a hybrid eigenbrain-based CAD system that can not only detect AD from NC, but also detect brain regions that related to AD. (iii) We proved the proposed method had classification accuracy comparable to state-of-the-art methods, and the detected brain regions were in line with 16 existing literatures. (iv) We used inter-class variance (ICV) and Welch's *t*-test (WTT) to reduce redundant data; (v) We found POL kernel is better than linear and RBF kernel for this study.

## Literature review

In common convention, the automatic classification consisted of two stages: feature extraction and classifier construction. We reviewed over ten literatures, and analyzed themthrough the two stages.

### Features of MR images

Scholars have proposed numerous methods to extract various features^1^. Chaplot et al. (2006) used the approximation coefficients obtained by discrete wavelet transform (DWT). Maitra and Chatterjee (2006) employed the Slantlet transform, which is an improved version of DWT. Their feature vector of each image was created by considering the magnitudes of Slantlet transform outputs corresponding to six spatial positions that were chosen according to a specific logic. El-Dahshan et al. (2010) extracted the approximation and detail coefficients of 3-level DWT. Plant et al. (2010) used brain region cluster (BRC). They suggested to use information gain (IG) to rate the interestingness of a voxel, and applied clustering algorithm to identify groups of adjacent voxels with a high discriminatory power. Zhang et al. (2011) exclusively used the approximation coefficients of 3-level decomposition, and used PCA to reduce the features. Ramasamy and Anandhakumar (2011) used fast Fourier transform (FFT) as features. Saritha et al. (2013) proposed a novel feature of wavelet-entropy, and employed spider-web plots to further reduce features. Zhang et al. (2013) employed digital wavelet transform to extract features then used principal component analysis (PCA) to reduce the feature space. Savio and Grana (2013) proposed to use deformation-based morphometry (DBM) techniques, and proposed five features as Jacobian map, modulated GM (MGM), trace of Jacobian matrix (TJM), magnitude of the displacement field, and Geodesic Anisotropy (GEODAN). In addition, they suggested the use of Pearson's correlation (PEC), Bhattacharyya distance (BD), and WTT to measure the significance of voxel site. Das et al. (2013) suggested to use Ripplet transform, followed by PCA to reduce features. Kalbkhani et al. (2013) modeled the detail coefficients of 2-level DWT by generalizing autoregressive conditional heteroscedasticity (GARCH) statistical model, and the parameters of GARCH model were considered as the primary feature vector. Zhang et al. (2014) used an undersampling (US) technique on the volumetric image, followed by singular value decomposition (SVD) to select features. El-Dahshan et al. (2014) proposed to add a preprocessing technique that used pulse-coupled neural network (PCNN) for image segmentation. Zhou et al. (2015) used wavelet-entropy as the feature space. Zhang et al. (2015a) used discrete wavelet packet transform (DWPT), and harnessed Tsallis entropy to obtain features from DWPT coefficients. Yang et al. (2015) selected wavelet-energy as the features.

From the literature used, the DWT based features were proven to be efficient. In this study, we suggested using a novel feature of eigenbrain, which was used for SPECT images but was never been used in MR images.

### Classification model in MRI

There are numerous classification models, but only a few of them are suitable for MR images. Chaplot et al. (2006) employed the self-organizing map (SOM) neural network and SVM. Maitra and Chatterjee (2006) used the common artificial neural network (ANN). El-Dahshan et al. (2010) used ANN and K-nearest neighbor (KNN) classifiers. Plant et al. (2010) used SVM, Bayes statistics, and voting feature intervals (VFI) to derive the quantitative index of pattern matching. Zhang et al. (2011) suggested to use ANN. The weights of ANN were trained by scaled-conjugate-gradient method. Ramasamy and Anandhakumar (2011) proposed to use Expectation and Maximization Gaussian Mixture Model algorithm (EM-GMM). Saritha et al. (2013) used the probabilistic neural network (PNN). Zhang et al. (2013) constructed a kernel SVM with RBF kernel, using particle swarm optimization (PSO) to optimize the parameters *C* and sigma. Savio and Grana (2013) chose SVM, and used grid search for tuning parameters. Das et al. (2013) used least-square SVM, and their 5 × 5 CV showed high classification accuracy. Kalbkhani et al. (2013) tested the KNN and SVM models. Zhang et al. (2014) proposed to combine KSVM and decision tree, and their method was dubbed KSVM-DT. El-Dahshan et al. (2014) used feed forward back-propagation neural network (FFBPNN). Zhou et al. (2015) used a Naive Bayes classifier (NBC) as classification method. Zhang et al. (2015a) used a generalized eigenvalue proximal SVM (GEPSVM) with RBF kernel. Yang et al. (2015) used SVM as the classifier, and employed biogeography-based optimization (BBO) to train the classifier.

After reviewing the latest literatures that were related to classifiers, we found that SVMs had significant advantages of high accuracy, elegant mathematical tractability, and direct geometric interpretation, compared with other classification methods (Collins and Pape, 2011). In addition, it did not need a large number of training samples to avoid overfitting (Li et al., 2010). Kernel technique further enhanced the performance of SVM. Therefore, KSVM was harnessed in this study.

## The proposed method

### Preprocessing on volumetric data

For each individual, all available 3 or 4 volumetric 3D MR brain images were motion-corrected, and coregistered to form an averaged 3D image. Then, those 3D images were spatially normalized to the Talairach coordinate space and brain-masked. CDR was interpreted as the target (label). It is a numeric scale quantifying the severity of symptoms of dementia (Williams et al., 2013). The patient's cognitive and functional performances were assessed in six areas: memory, orientation, judgment and problem solving, community affairs, home and hobbies, and personal care. In this study, we chose two types of CDR, i.e., the subjects with CDR of 0 were considered as NC and subjects with CDR of 1 were considered as AD (Marcus et al., 2007).

Calculating eigenbrains on the entire brain was difficult. Instead, we proposed a simplified method that selected several key slices that capture structures indicative of AD from NC. The procedure was as follows: we established the ICV *v* as

(1) $$v\left(k\right)={\|\mu_{\text{AD}}\left(Slice=k\right)-\mu_{\text{NC}}\left(Slice=k\right)\|}^{2}$$

where *k* was the index of key slice, μ_*AD*_ and μ_*NC*_ represented the mean of gray-level values of the *k*th slice of AD subjects and NC subjects, respectively, ||.||^2^ represented the *l*_2_-norm. Then, we selected the key-slices of ICV larger than 50% of maximum ICV, with 10× undersampling factor (i.e., every 10 slices).

**Figure 1:**
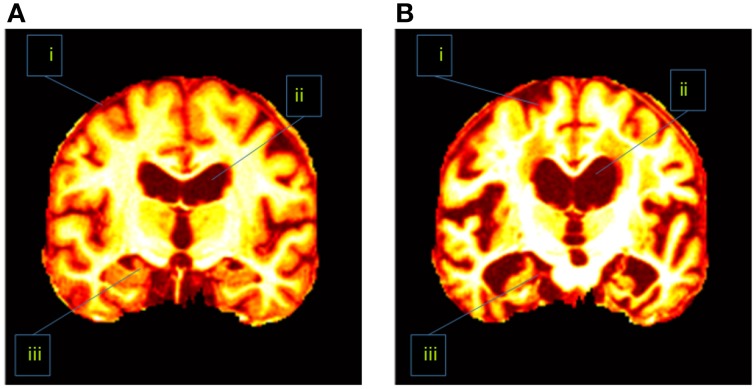
**Difference between (A) a healthy brain and (B) an AD brain**. The labeled three regions are (i) cerebral cortex (ii) ventricle, and (iii) hippocampus.

In addition, the slice direction can be chosen as either axial, sagittal, or coronal. Usually coronal direction will give a clearer view than the other two directions. Figure 1 showed that the coronal slice had an advantage over other directions in that it can cover three of the most important tissues within one slice. Those tissues were seen as indicative of AD. These tissues are the cerebral cortex, the ventricle, and the hippocampus. If we used axial or sagittal slice, then we may need to record two or even more slices to cover those tissues. Therefore, we chose the coronal direction for key slice selection, with the aim of recording only one slice.

### Eigenbrain

AD has different physical structures from NC. Revisit Figure 1 which indicated the AD subjects had severe atrophy of the cerebral cortex (region i), severely enlarged ventricles (region ii), and extreme shrinkage of hippocampus (region iii). Therefore, eigenbrain tried to capture those different characteristic changes of anatomical structures between AD and NC.

Eigenbrain is carried out by PCA, which is a statistical procedure that uses an orthogonal transformation to convert a set of observations of possibly correlated variables into a set of values of linearly uncorrelated variables called principal components (PC). For 2D images the PCs are extended naturally to the 2D eigenbrains.

Suppose ***X*** is a given data matrix with size of *N* × *A*, where *N* represents the number of samples and *A* number of attributes (For a 256 × 256 image, we need to vectorize it to a 1 × 65536 vector, hence *A* = 65536). First, we normalized the dataset matrix ***X***, so that each sample in the normalized matrix ***Z*** was mean-centered and unit-variance scaled, by subtracting its mean value and dividing the difference by its standard deviation.

(2) $$Z\leftarrow \frac{X-\mu \left(X\right)}{\sigma \left(X\right)}$$

Next, we estimated the covariance matrix ***C*** with size of *A* × *A* by

(3) $$C\leftarrow \frac{1}{N-1}Z^{T}Z$$

Here we used *N* − 1 instead of *N* in order to produce an unbiased estimator of the variance (See Bessel's correction (Russell and Cohn, 2012) for details).

Third, we perform the eigendecomposition of ***C***:

(4) $$C=U\wedge U^{-1}$$

where ***U*** is an *A* × (*N* − 1) matrix, whose columns are the eigenvectors of covariance matrix ***C***, matrix Λ is an (*N* − 1) × (*N* − 1) diagonal matrix whose diagonal elements are eigenvalues of ***C***, each corresponding to an eigenvector of *A*. It is common to sort the eigenvalue matrix Λ and eigenvector matrix ***U*** in order of decreasing eigenvalue *u*_1_ > *u*_2_ > … > *u_N_*. To view the *i*th eigenbrain *u*(*i*), the *i*th column of ***U*** was reshaped to an image. Suppose the *i*th column of ***U*** contains 65536 elements, then the reshaped image was 256 × 256.

(5) u(i)=reshape(U(:,i))

Note that in our situation (*N* ~ 10^2^ and *A* ~ 10^4^, where ~ denotes the same order of magnitude), the computation burdens of eigendecomposition of equation (4) are enormous. It can be accelerated by replacing ***C*** in equation (3) with ***C***′, since *N* << *A*.

(6) $$C^{\prime }\leftarrow \frac{1}{N-1}ZZ^{T}$$

**Figure 2:**
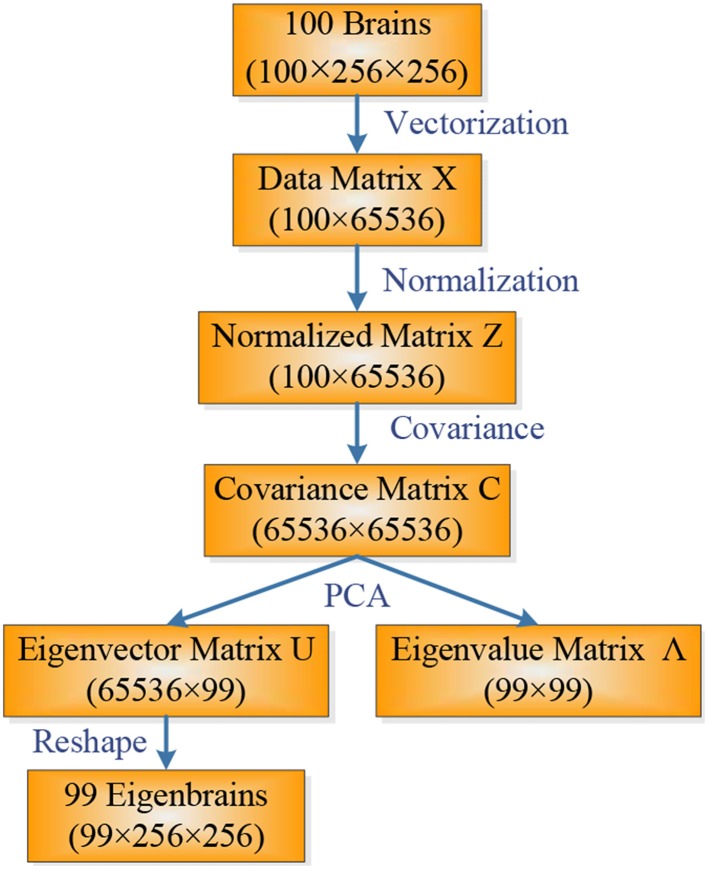
**Flowchart of calculating eigenbrain**.

The size of ***C***′ is *N* × *N*, which can significantly reduce the computation burden. Using Matlab, the eigenbrain can be done by a simple “PCA” command without considering these issues. The flowchart of calculating eigenbrain is shown in Figure 2.

The eigenvalues represent the distribution of energy of the source data among each of the eigenbrains, where the eigenbrains form a basis for original data.

To further select an eigenbrain that is the most statistically significant, we employ the two-sample location test. Saritha et al. (2013) selected the Student's *t*-test which assumes both the means and variances of the two data are equal. The assumption of equal variances was not necessary and can be dropped; while the assumption of equal means is essential to select significantly important eigenbrains. Therefore, we used WTT that is an adaption of the Student's *t*-test and checks nothing except the two populations that have equal means.

The null hypothesis is that the eigenvalues of AD and NC have equal means, without assuming they have equal variances. The alternative hypothesis is they have unequal means. WTT was carried out at the 95% confidence interval. The eigenvalues of the selected most important eigenbrain (MIE) were used as input features for following classification.

### Region detection

We proposed a visual interpretation method of Eigenbrain to detect regions that can distinguish AD and NC, which is not reported in literatures of Alvarez et al. (2009a) and Lopez et al. (2009). The interpretation in a four-stage process is listed in Table 1.

**Table 1:** **Four-stage region detection method**.

**Region detection**
Step 1 We selected the most important eigenbrain (MIE).
Step 2 We performed an absolution operation on MIE, since there are both positive and negative elements in the MIE matrix.
Step 3 We highlighted those voxels with the values higher than 0.98 quantile, i.e., 98th percentile.
Step 4 We outputted the anatomical label information of selected voxels using Talairach Daemon software, the output of which contained five levels: hemisphere, lobe, gyrus, tissue, and cell.

### Classifier

SVM was used as the classifier. In addition, sequential minimal optimization (SMO) is chosen to train SVM due to simple and fast speed (Zhang and Wu, 2012b). Traditional linear SVMs cannot separate intricately distributed data. In order to generalize SVMs to create nonlinear hyperplane, the kernel trick is applied. The KSVMs allows us to fit the maximum-margin hyperplane in a transformed feature space (Liu et al., 2014). The transformation may be nonlinear and the transformed space is a higher dimensional space. Though the classifier is a hyperplane in the higher-dimensional feature space, it may be nonlinear in the original input space.

The radial basis function (RBF) kernel is one of the most widely used kernels with the form as Zhang and Wu (2012b).

(7) $$\kappa \left(x_{m},x_{n}\right)=\text{exp}\left(-\frac{\|x_{m}-x_{n}\|}{2\sigma^{2}}\right)$$

where κ is the kernel function, σ the scaling factor, and *x_m_* and *x_n_* are vectors in the input space.

Another commonly used kernel is polynomial (POL) kernel defined as

(8) $$\kappa \left(x_{m},x_{n}\right)={\left(x_{m}^{T}x_{n}+c\right)}^{d}$$

where *d* is the degree of polynomial, and *c* a soft margin constant trading off the influence of higher-order vs. lower-order terms in the polynomial.

Based on the two kernels, we tested RBF-KSVM and POL-KSVM for our models. To obtain the best parameter of kernels (the scaling factor σ of RBF, or the degree *d* and soft margin constant *c* of POL), PSO was employed since it has been used successfully to tune parameters of KSVM in various problems (Aich and Banerjee, 2014; Khazaee and Zadeh, 2014; Xue et al., 2014).

*K*-fold CV was employed, and K was assigned with a value of 10 considering the best compromise between computational cost and reliable estimates, i.e., the dataset is randomly divided into 10 mutually exclusively subsets of approximately equal size, in which 10 − 1 = 9 subsets were used as training set and the last subset was used as the validation set. The procedure that was mentioned above was repeated 10 times, so each subset was used once for validation. The K results from the K folds were combined together, to yield a single estimation of the whole dataset.

The *K*-fold CV repeated 50 times, i.e., we carried out a 50 × 10-fold CV. For each time, we used four measures: accuracy, sensitivity, specificity, and precision (Table 2), to assess the performance. Here TP, FP, TN, and FN represented the instance number of true positive, false positive, true negative, and false negative, respectively. We considered a correctly identified AD case as a true positive, following the common convention. Summarizing the 50 repetitions, we reported the final measures of both the mean and standard deviation (SD) of the four measures.

**Table 2:** **Assessment of classification performance**.

**Measure**	**Definition**
Accuracy	(*TP* + *TN*) / (*TP* + *TN* + *FP* + *FN*)
Sensitivity (Recall)	*TP* / (*TP* + *FN*)
Specificity	*TN* / (*TN* + *FP*)
Precision	*TP* / (*TP* + *FP*)

### Implementation

The purpose of the proposed method is two-fold: (i) to find discriminant voxels that distinguish AD from NC; and (ii) to develop a CAD system and report its performance. The pseudocode is listed in Table 3.

**Table 3:** **Pseudocode of proposed method**.

**Step 1** Input 3D MRI data and corresponding CDR labels.
**Step 2** Select key slices by ICV larger than 50% of maximum, with 10× undersampling factor.
**Step 3** Generate eigenbrain set for each key slice.
**Step 4** Select the MIE by WTT with 95% confidence interval.
**Step 5** (**Output 1**): Submit eigenvalues of MIE to the classifier, and report its performance based on 50 × 10 CV.
**Step 6** (**Output 2**): Report the discriminant regions by the absolute coefficient values higher than 0.98 quantile.

## Experiments and results

The programs were in-house developed using Matlab 2014a, and ran on IBM laptop with 3 GHz Intel i3 dual-processor and 8 GB RAM. Readers could repeat our results on any machine where Matlab is available.

### Data source

We downloaded the dataset from Open Access Series of Imaging Studies (OASIS) (Ardekani et al., 2013, 2014). We chose the cross-sectional dataset corresponding to MRI scans of individuals at a single time point (Bin Tufail et al., 2012). The OASIS dataset consists of 416 subjects aged 18–96, who are all right-handed. We excluded subjects under 60 years old and those with missing records and then picked 126 subjects (98 NCs and 28 ADs) from the rest of the subjects. The demographic statuses of the included subjects were summarized in Table 4. Here SES, CDR, and MMSE represent socioeconomic status, clinical dementia rating, and mini-mental state examination, respectively.

**Table 4:** **Subject demographics status**.

	**NC**	**AD**
Number of subjects	98	28
Male/Female	26/72	9/19
Age	75.91 ± 8.98	77.75 ± 6.99
Education	3.26 ± 1.31	2.57 ± 1.31
SES	2.51 ± 1.09	2.87 ± 1.29
CDR	0	1
MMSE	28.95 ± 1.20	21.67 ± 3.75

### Preprocessing

Table 5 shows an example of the combination of 3 individual scans of a subject. The resolution is 1 × 1 × 1.25 mm. The preprocessing performed motion-correction on the 3D MR images, registered them to form a combined image in the native acquisition space, and resampled to 1 × 1 × 1 mm. Afterwards, the combined image was spatially normalized to the Talairach coordinate space, and brain-extracted (Table 5).

**Table 5:**
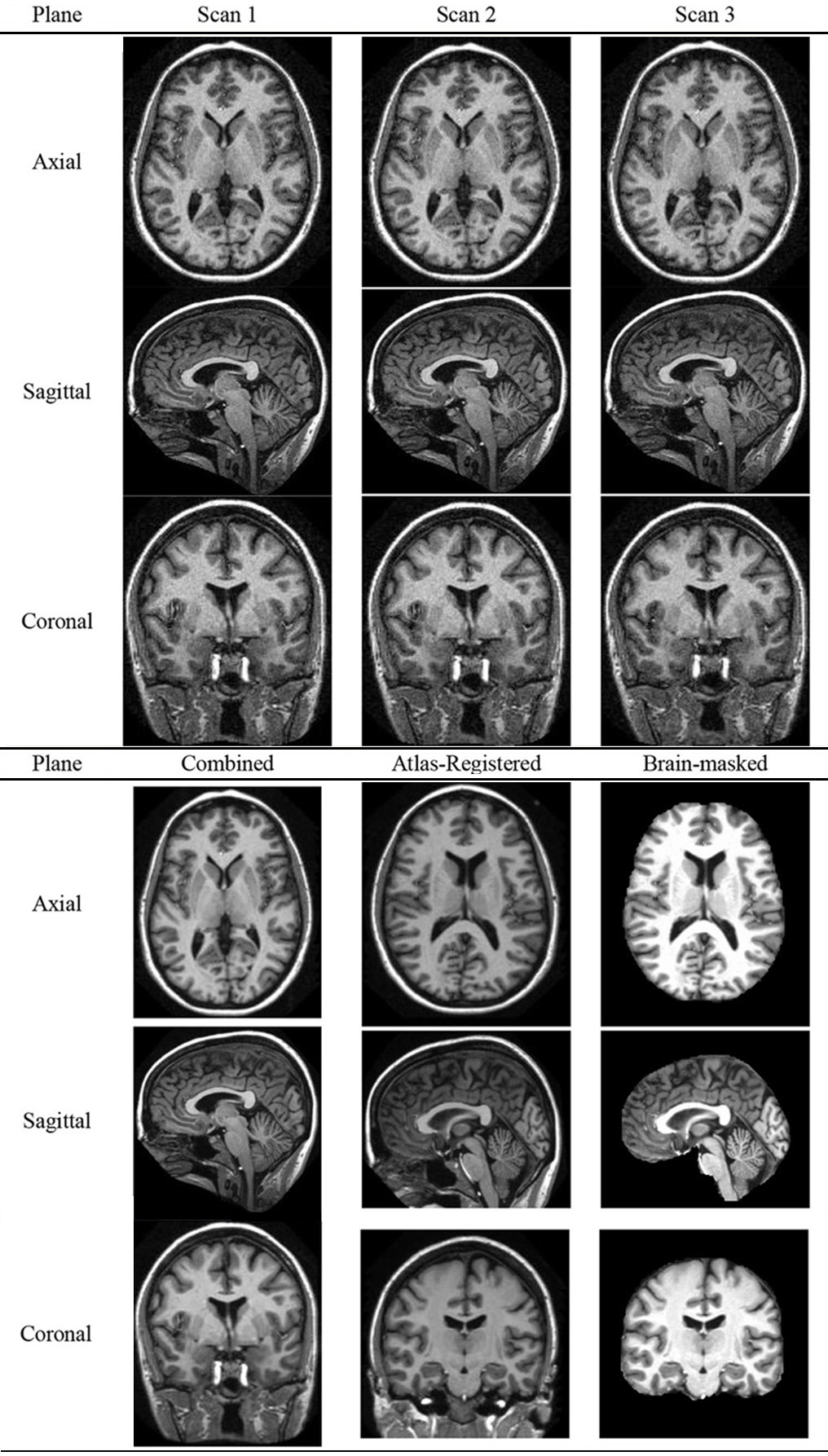
**Preprocessing of a specified subject**.

### Key-slice selection by ICV

**Figure 3:**
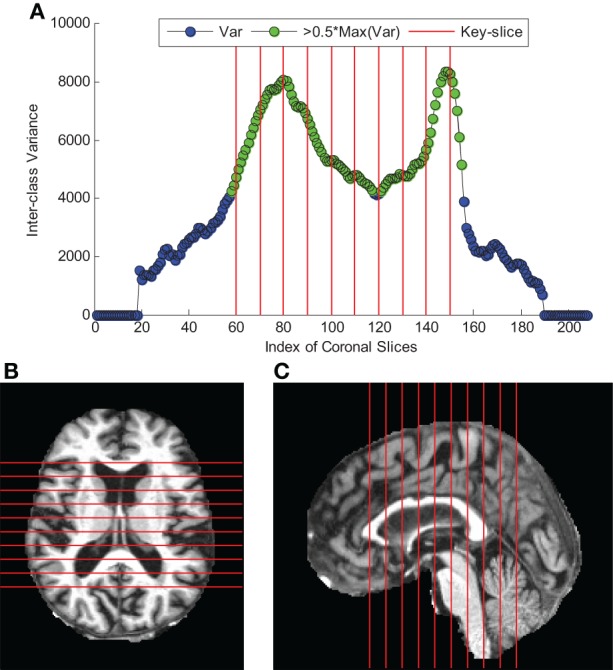
**Key-Slice selection (The red lines correspond to key-slices). (A)** The curve of ICV against coronal slice index. **(B)** axial view of key-slices. **(C)** sagittal view of key-slices.

The curve of ICV against slice index was shown in Figure 3A. We selected 10 coronal slices (60, 70, 80, 90, 100, 110, 120, 130, 140, and 150). Their corresponding ICVs were all higher than 50% of the maximum. Figures 3B,C showed the axial and sagittal view of the 10 key-slices. Table 6 showed the comparison between NC and AD in the selected 10 key-slices.

**Table 6:**
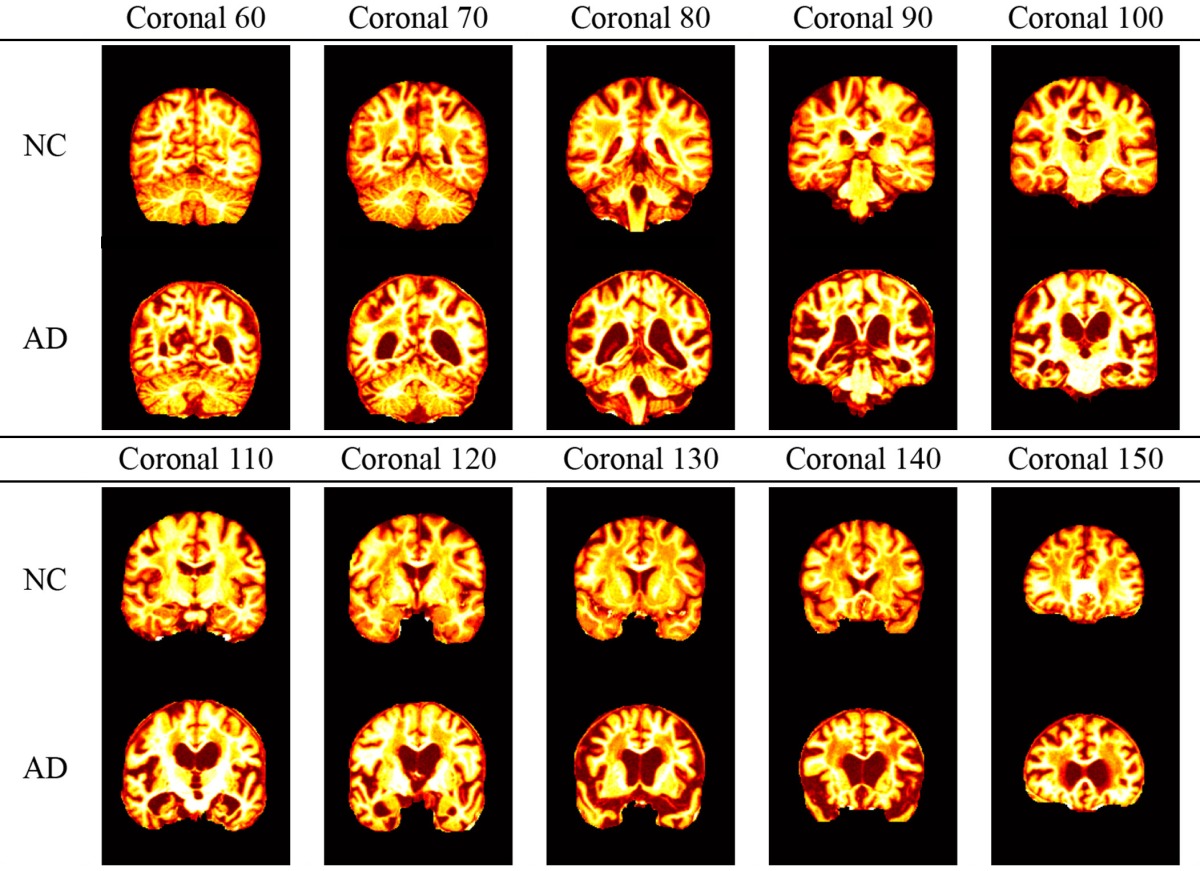
**Difference between NC and AD on key-slices**.

### Eigenbrains

Table 7 showed the eigenbrain results obtained by running PCA on the slices of all subjects. For each slice, we had a set of 125 eigenbrains in total. Due to the page limit, we selected and listed the first 6 eigenbrains. The eigenbrains were sorted in the order of decreasing eigenvalues. In general, the eigenbrains in the previous columns were more important than in latter columns.

**Table 7:**
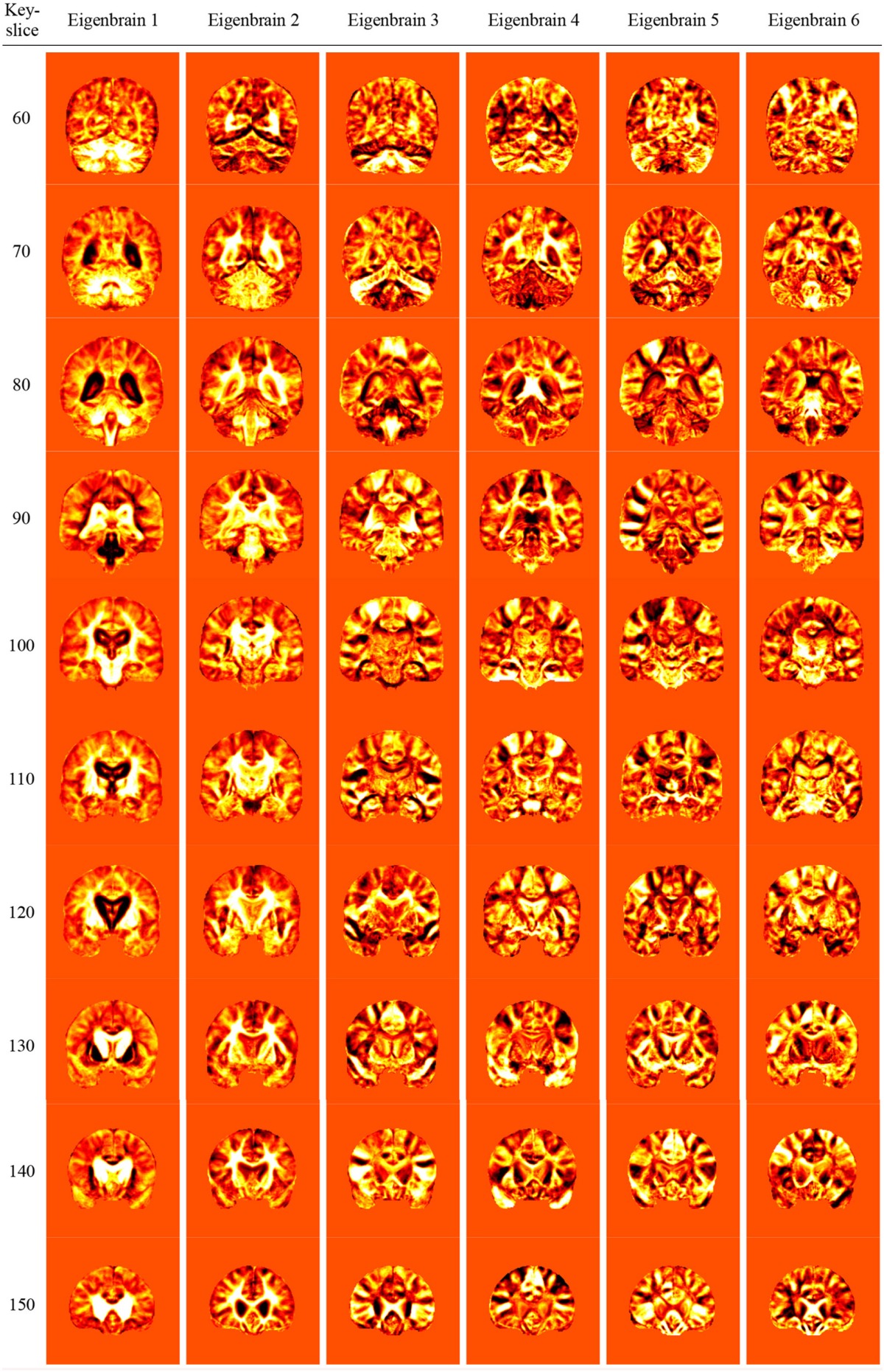
**Eigenbrain results**.

### Most important eigenbrain

WTT was conducted to give quantified proof of why the first eigenbrain was MIE. We performed WTT for the first six eigenbrains of all key-slices between eigenvalues to characterize those that were AD and those that were NC. The results were shown in Table 8, and *p*-values less than 0.05 were marked in bold. Only the first eigenvalues of all slices were less than 0.05; therefore, the first eigenbrain was indeed the MIE, and we assigned the eigenvalues of MIE of all 10 key-slices (namely, 10 × 1 = 10 features) of each subject to classification.

**Table 8:** **WTT of the first six eigenvalues of 10 key-slices**.

	**λ_1_**			**λ_2_**			**λ_3_**		
**Slice**	**NC**	**AD**	**p**	**NC**	**AD**	**p**	**NC**	**AD**	**p**
60	−3.36 ± 20.01	11.75 ± 27.91	**0.01**	2.82 ± 18.77	−9.87 ± 27.93	**0.03**	0.11 ± 18.95	−0.39 ± 21.44	0.91
70	−6.84 ± 25.60	23.92 ± 28.33	**0.00**	0.43 ± 21.20	−1.50 ± 36.97	0.79	1.84 ± 19.88	−6.44 ± 22.86	0.09
80	−7.48 ± 29.05	26.18 ± 27.04	**0.00**	−0.65 ± 22.00	2.26 ± 33.36	0.67	−0.25 ± 21.84	0.87 ± 25.08	0.83
90	6.79 ± 32.04	−23.75 ± 24.86	**0.00**	0.42 ± 21.94	−1.46 ± 32.98	0.78	−1.88 ± 20.16	6.57 ± 21.48	0.07
100	−6.93 ± 34.25	24.27 ± 30.89	**0.00**	2.51 ± 23.05	−8.79 ± 31.63	0.09	0.63 ± 20.16	−2.22 ± 23.74	0.57
110	−6.95 ± 31.89	24.31 ± 24.10	**0.00**	0.48 ± 25.03	−1.67 ± 32.93	0.75	1.95 ± 18.17	−6.81 ± 29.05	0.14
120	−5.93 ± 31.60	20.74 ± 23.14	**0.00**	−0.33 ± 24.02	1.14 ± 31.84	0.82	−1.07 ± 16.73	3.74 ± 25.61	0.35
130	5.02 ± 28.13	−17.56 ± 28.09	**0.00**	−1.40 ± 21.70	4.90 ± 27.75	0.27	−0.59 ± 17.75	2.06 ± 19.20	0.52
140	4.27 ± 25.02	−14.94 ± 22.06	**0.00**	−1.34 ± 18.13	4.70 ± 27.10	0.27	3.12 ± 17.91	−10.93 ± 14.69	**0.00**
150	5.51 ± 18.50	−19.30 ± 30.21	**0.00**	−2.22 ± 18.08	7.78 ± 24.66	0.05	1.42 ± 16.56	−4.97 ± 13.98	0.05
	**λ_4_**			**λ_5_**			**λ_6_**		
**Slice**	**NC**	**AD**	**p**	**NC**	**AD**	**p**	**NC**	**AD**	**p**
60	−1.27 ± 15.47	4.43 ± 25.32	0.27	1.51 ± 14.13	−5.29 ± 23.59	0.16	−1.29 ± 13.10	4.50 ± 23.71	0.22
70	1.99 ± 17.76	−6.95 ± 22.50	0.06	−0.03 ± 16.69	0.09 ± 23.25	0.98	−0.96 ± 16.08	3.35 ± 20.79	0.32
80	1.46 ± 21.14	−5.12 ± 18.85	0.12	−0.72 ± 17.80	2.52 ± 24.31	0.51	−1.34 ± 17.47	4.68 ± 21.78	0.19
90	0.31 ± 19.66	−1.09 ± 23.73	0.78	−0.54 ± 18.05	1.89 ± 24.49	0.63	−1.80 ± 16.79	6.29 ± 23.33	0.10
100	−1.56 ± 18.77	5.47 ± 21.18	0.12	0.84 ± 16.32	−2.95 ± 25.35	0.46	−0.53 ± 15.58	1.85 ± 24.87	0.63
110	−0.31 ± 19.32	1.07 ± 17.30	0.72	0.54 ± 16.78	−1.87 ± 22.19	0.60	−1.09 ± 16.07	3.83 ± 20.43	0.25
120	−0.32 ± 16.83	1.13 ± 21.16	0.74	−2.21 ± 18.00	7.74 ± 10.70	**0.00**	−1.31 ± 14.81	4.57 ± 21.45	0.18
130	1.61 ± 17.00	−5.62 ± 18.51	0.07	1.39 ± 14.21	−4.86 ± 23.47	0.19	2.01 ± 15.42	−7.04 ± 17.25	**0.02**
140	2.11 ± 16.81	−7.39 ± 16.29	**0.01**	0.44 ± 15.37	−1.56 ± 17.70	0.59	1.21 ± 14.37	−4.24 ± 17.85	0.15
150	1.17 ± 13.52	−4.11 ± 18.51	0.17	0.27 ± 14.35	−0.94 ± 13.89	0.69	0.17 ± 13.52	−0.58 ± 15.14	0.82

*P-values less than 0.05 are in bold*.

### Classification comparison

The two classes in order were AD and NC, following common convention. Here we designed three tasks. The first did not use the kernel technique, i.e., the basic linear SVM; the second used RBF-KSVM; and the third used POL-KSVM. The kernel parameters and error penalty were optimized by PSO method. The classification results were listed in Table 9, in addition with the results of state-of-the-art methods.

**Table 9:** **Comparison of classification results**.

	**Accuracy**	**Sensitivity**	**Specificity**	**Precision**
**EXISTING METHODS**				
US + SVD-PCA + SVM-DT (Zhang et al., 2014)	90	94	71	N/A
BRC + IG + SVM (Plant et al., 2010)	90.00 [77.41, 96.26]	96.88 [82.01, 99.84]	77.78 [51.92, 92.63]	N/A
BRC + IG + Bayes (Plant et al., 2010)	92.00 [79.89, 97.41]	93.75 [77.78, 98.27]	88.89 [63.93, 98.05]	N/A
BRC + IG + VFI (Plant et al., 2010)	78.00 [63.67, 88.01]	65.63 [46.78, 80.83]	100.00 [78.12, 100]	N/A
MGM + PEC + SVM (Savio and Grana, 2013)	92.07 ± 1.12	86.67 ± 4.71	N/A	95.83 ± 5.89
GEODAN + BD + SVM (Savio and Grana, 2013)	92.09 ± 2.60	80.00 ± 4.00	N/A	88.09 ± 5.33
TJM + WTT + SVM (Savio and Grana, 2013)	92.83 ± 0.91	86.33 ± 3.73	N/A	85.62 ± 0.85
**PROPOSED METHODS**				
ICV + Eigenbrain + WTT + SVM	91.47 ± 1.02	90.17 ± 1.66	91.84 ± 1.09	93.21 ± 2.43
ICV + Eigenbrain + WTT + RBF-KSVM	86.71 ± 1.93	85.71 ± 1.91	86.99 ± 2.30	66.12 ± 4.16
ICV + Eigenbrain + WTT + POL-KSVM	92.36 ± 0.94	83.48 ± 3.27	94.90 ± 1.09	82.28 ± 2.78

### Region detection

We carried out the region detection procedure from MIE as Section Region Detection described. Table 10 showed the result, in which the green points represented the discriminant voxels.

**Table 10:**
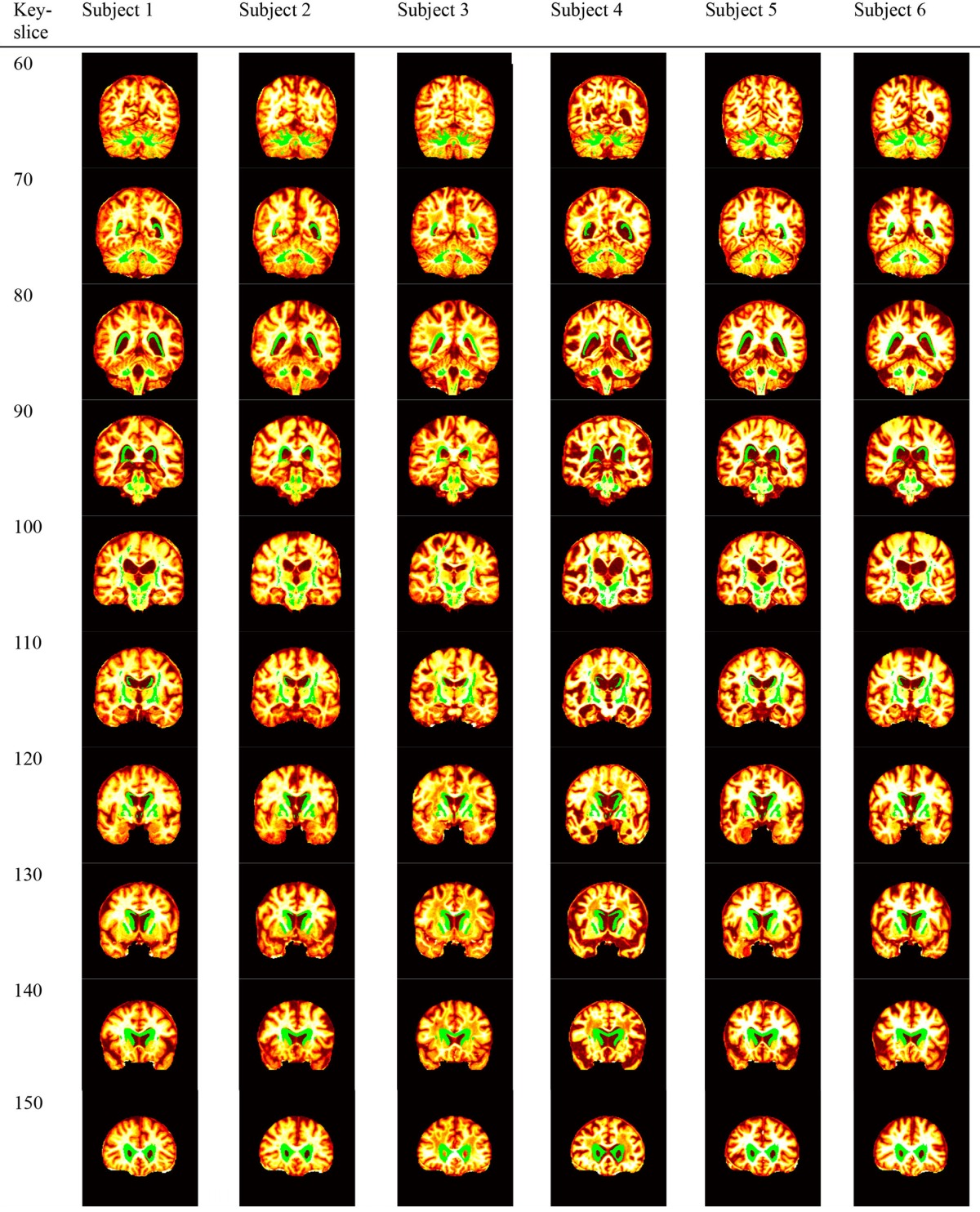
**Discriminant voxels**.

Here we reported the discriminative regions interpreted by eigenbrain in Table 11, where BA represented Brodmann area.

**Table 11:** **Regions found by Eigenbrain**.

**Regions**	**# of voxels**	**Reported by**
Anterior cingulate (BA-24, BA-32)	35	Schultz et al., 2014
Caudate nucleus (Head, body, and tail)	407	Möller et al., 2015
Cerebellum	65	Colloby et al., 2014
Cingulate gyrus (BA-23, BA-24, BA-31)	343	Yu et al., 2014
Claustrum	14	De Reuck et al., 2014
Inferior frontal gyrus (BA-47)	71	Eliasova et al., 2014
Inferior parietal lobule (BA-40)	29	Wang et al., 2015
Insula (BA-13)	23	He et al., 2015
Lateral ventricle	410	Voevodskaya et al., 2014
Lentiform nucleus	569	Möller et al., 2015
Lingual gyrus	71	Lehmann et al., 2013
Medial frontal gyrus (BA-10, BA-11, BA-25, BA-6)	416	Kang et al., 2013
Middle frontal gyrus (BA-11)	52	Schultz et al., 2014
Middle occipital gyrus	22	Lehmann et al., 2013
Middle temporal gyrus	50	Aubry et al., 2015
Paracentral lobule (BA-3, BA-4, BA-5, BA-6, BA-7)	210	Kang et al., 2013
Parahippocampal gyrus (Amygdala, BA-28, BA-35, Hippocampus)	276	Eskildsen et al., 2015
Postcentral gyrus (BA-5)	10	Kang et al., 2013
Posterior cingulate	27	Shinohara et al., 2014
Precentral gyrus (BA-4)	11	Kang et al., 2013
Precuneus (BA-7, BA-31)	557	Kang et al., 2013
Subcallosal gyrus (BA-25, BA-34, BA-47)	82	Paakki et al., 2010
Sub-Gyral (BA-40, Corpus Callosum, Hippocampus)	589	Streitburger et al., 2012
Superior frontal gyrus	70	Chen et al., 2014
Superior parietal lobule	269	Quiroz et al., 2013
Superior temporal gyrus (BA-38)	12	Paakki et al., 2010
Supramarginal gyrus	14	Quiroz et al., 2013
Thalamus (Medial Geniculum Body, Pulvinar, Ventral Lateral Nucleus)	35	He et al., 2015
Transverse Temporal Gyrus (BA-41)	26	Kim et al., 2012
Uncus (BA-28)	25	Bangen et al., 2014

## Discussion

It is clearly observed in Table 6 that the selected coronal slices are significant in detecting AD from NC. In particular, the AD subjects show the cerebrospinal fluid (CSF) in the areas occupied by brain matter in the NC subjects. We conclude that 10× is reasonable because of following three reasons: (1) The 10× key-slice undersampling (i.e., select only one slice from 10 consecutive slices) yields a coarser brain while still capturing most tissues in the brain (Compare Table 6 with Figure 1). (2) It is very hard to define a fitness (optimization) function to find the optimal undersampling factor. (3) The classification system has a good accuracy in distinguishing AD from NC, and it detects correct AD-related brain regions (See Tables 9, 11). As there are spatial redundancy for neighboring coronal slices, the undersampling could reduce this redundancy to a rather small degree.

Overall, the eigenbrains in Table 7 capture both similarities and differences of structural features between AD and NC. The first eigenbrain capture the significant feature of AD from NC, and the second and following eigenbrains capture general brain structure. Revisiting the hippocampus part in the first eigenbrain of all key-slices, it is easily perceived that the body lateral ventricles area of AD are highlighted, which is indeed a distinct attribute between AD and NC. Our experiment extends the eigenbrain on SPECT images by Alvarez et al. (2009a) and Lopez et al. (2009) and shows that eigenbrain is also suitable for MRI scans.

The *p*-values in Table 8 show that the first eigenvalue λ_1_ are all less than 0.05 for all key-slices. It indicates that mean values of λ_1_ of AD and NC are significantly different. Hence, the most dominating eigenvalue characterizing AD and NC is the one corresponding to the first eigenbrain. For other eigenvalues, merely 1 of 10 *p*-values is less than 0.05, which indicates that those eigenbrains are not dominating features indicative of AD from NC. Therefore, the first eigenvalue is MIE and was selected.

Classification results in Table 9 compare the proposed three classifiers with state-of-the-art methods, in which Zhang's results (Table 7 in Zhang et al., 2014) are calculated through a single *K*-fold CV experiment. Plant's results (Task 1 in Table 3 Plant et al., 2010) offer the means together with 95% confidence intervals. Savio's results (Table 5 Savio and Grana, 2013) give the means with SD. For the proposed methods, it is **unexpected** that the POL-KSVM produces better classification accuracy of 92.36 ± 0.94 than linear SVM of 91.47 ± 1.02 and RBF-KSVM of 86.71 ± 1.93, because RBF was reported as the most widely used kernel. Our results are better than or comparable to other approaches to AD prediction from MR brain images of NC, e.g., US + SVD-PCA + SVM-DT of 90% (Zhang et al., 2014), BRC + IG + SVM of 90% (Plant et al., 2010), BRC + IG + Bayes of 92% (Plant et al., 2010), MGM + PEC + SVM of 92.07% (Savio and Grana, 2013), GEODAN + BD + SVM of 92.09% (Savio and Grana, 2013), and TJM + WTT + SVM of 92.83% (Savio and Grana, 2013). There were many other methods (Gray et al., 2012; Arbizu et al., 2013; Chaves et al., 2013; Dukart et al., 2013; Cohen and Klunk, 2014) proposed for detecting AD from NC, however, they treated images from other modalities (such as SPECT and PET). Therefore, it is not appropriate to compare the proposed methods with them. We will test our methods on SPECT and PET images in the future.

Table 11 shows that eigenbrains interpret the discriminant voxels involving the following regions reported in existing literatures: Anterior Cingulate (BA-24, BA-32) (Schultz et al., 2014), Caudate Nucleus (Head, body, and tail) (Möller et al., 2015), Cerebellum (Colloby et al., 2014), Cingulate Gyrus (BA-23, BA-24, BA-31) (Yu et al., 2014), Claustrum (De Reuck et al., 2014), Inferior Frontal Gyrus (BA-47) (Eliasova et al., 2014), Inferior Parietal Lobule (BA-40) (Wang et al., 2015), Insula (BA-13) (He et al., 2015), Lateral Ventricle (Voevodskaya et al., 2014), Lentiform Nucleus (Möller et al., 2015), Lingual gyrus (Lehmann et al., 2013), Medial Frontal Gyrus (BA-10, BA-11, BA-25, BA-6) (Kang et al., 2013), Middle Frontal Gyrus (BA-11) (Schultz et al., 2014), Middle Occipital Gyrus (Lehmann et al., 2013), Middle Temporal Gyrus (Aubry et al., 2015), Paracentral Lobule (BA-3, BA-4, BA-5, BA-6, BA-7) (Kang et al., 2013), Parahippocampal Gyrus (Amygdala, BA-28, BA-35, Hippocampus) (Eskildsen et al., 2015), Postcentral Gyrus (BA-5) (Kang et al., 2013), Posterior Cingulate (Shinohara et al., 2014), Precentral Gyrus (BA-4) (Kang et al., 2013), Precuneus (BA-7, BA-31) (Kang et al., 2013), Subcallosal Gyrus (BA-25, BA-34, BA-47) (Paakki et al., 2010), Sub-Gyral (BA-40, Corpus Callosum, Hippocampus) (Streitburger et al., 2012), Superior Frontal Gyrus (Chen et al., 2014), Superior Parietal Lobule (Quiroz et al., 2013), Superior Temporal Gyrus (BA-38) (Paakki et al., 2010), Supramarginal Gyrus (Quiroz et al., 2013), Thalamus (Medial Geniculum Body, Pulvinar, Ventral Lateral Nucleus) (He et al., 2015), Transverse Temporal Gyrus (BA-41) (Kim et al., 2012), and Uncus (BA-28) (Bangen et al., 2014).

Nevertheless, some regions reported to be associated with AD are not interpreted by Eigenbrain, such as subthalamic nucleus (De Reuck et al., 2014). The reason may lie in three aspects. First, the quantile of our method is assigned with a value of 0.98, which is considered high. Reducing the quantile value may include more regions. Second, some literature used other advanced imaging modalities, such as MRSI and fMRI for metabolism detection and function analysis. Third, the key-slice selection procedure may miss important regions.

From another point of view, Table 11 demonstrates the power of the eigenbrain. Our study uses only one feature (eigenbrain) on 10 key-slices of a simple 3D structural MR image, nevertheless, our findings cover 30 related regions reported by over twenty literatures, which used various feature extraction methods and advanced imaging technologies.

The **contributions** of the paper fall within the following five aspects: (i) We generalize the Eigenbrain to MR images, and prove its effectiveness; (ii) We propose a hybrid eigenbrain-based CAD system that can not only detect AD from NC, but also detect brain regions that related to AD. (iii) We prove the proposed method has a classification accuracy comparable to state-of-the-art methods, and the detected brain regions are in line with 16 existing literatures. (iv) We use ICV and WTT to reduce redundant data; (v) we find POL kernel is better than linear and RBF kernel for this study.

In conclusion, the advantages of eigenbrain are three-fold: (i) it reaches very high classification accuracy, which was better than or competitive with state-of-the-art methods (Plant et al., 2010; Savio and Grana, 2013; Zhang et al., 2014); (ii) it can directly find discriminant voxels/regions within the whole brain; (iii) it can be combined with other features, in order to increase the classification performance. On the other hand, the disadvantages of eigenbrain also exist: (i) it is essentially two-dimensional, which does not reduce the redundancy along the slice direction; (ii) it needs preprocessing of spatial registration, which costs large amount of computation resources.

To the policy-makers, this study suggests the eigenbrain technique can achieve comparable results to traditional methods. It may offer a ray of hope for AD diagnosis with unconventional means with the combination of eigenbrain and machine learning. This preclinical study suggests that hospitals and medical laboratories enroll more computer scientists and engineers, with the aim of developing efficient AD diagnosis and region detection systems.

## Conclusion and future research

We presented an automated and accurate classification method that was based on eigenbrains and machine learning, in order to detect AD subjects and AD-related brain regions using 3D MR images. The results showed the proposed POL-KSVM method achieved 92.36% accuracy, which was competitive with state-of-the-art methods.

In the future, we will focus our research in the following aspects: (i) We shall generalize the eigenbrain to three dimensional, so the procedure of key-slice selection can be removed; (ii) We shall test other kernels for SVM, and try to replace KSVM with other advanced pattern recognition tools. (iii) Eigenbrain can be used in combination with DWT-based features and others, and an increase in classification accuracy is expected.

### Conflict of interest statement

The authors declare that the research was conducted in the absence of any commercial or financial relationships that could be construed as a potential conflict of interest.

## Footnotes

^1^Some abbreviations are modified to avoid conflict within this paper.
