# Supplementary material for: Detection of subjects and brain regions related to Alzheimer's disease using 3D MRI scans based on eigenbrain and machine learning
Source: Front Comput Neurosci. 2015 Jun 2;9:66. doi: 10.3389/fncom.2015.00066 (PMC4451357; doi:10.3389/fncom.2015.00066)
Supplement: Supplementary file 1 [file Acronyms.DOCX]

# Appendix Acronyms

Table 12 List of Acronyms

| Acronym | Definition |
| --- | --- |
| (k)SVM | (kernel) support vector machine |
| AD | Alzheimer’s disease |
| ANN | artificial neural network |
| BA | Brodmann area |
| BD | Bhattacharyya distance |
| BRC | brain region cluster |
| CAD | computer-aided diagnosis |
| CDR | clinical dementia rating |
| CSF | Cerebrospinal fluid |
| CV | cross validation |
| DBM | deformation-based morphometry |
| DWT | discrete wavelet transform |
| GARCH | generalized autoregressive conditional heteroscedasticity |
| GEODAN | geodesic anisotropy |
| GEPSVM | generalized eigenvalue proximal SVM |
| ICV | Inter-Class variance |
| IG | information gain |
| KNN | K-nearest neighbor |
| MGM | Modulated GM |
| MIE | the Most Important Eigenbrain |
| MMSE | mini-mental state examination |
| MR(I) | magnetic resonance (imaging) |
| NBC | naive Bayes classifier |
| NC | normal elder controls |
| OASIS | Open Access Series of Imaging Studies |
| PC(A) | principal components (analysis) |
| PCNN | pulse-coupled neural network |
| PEC | Pearson’s correlation |
| PNN | probabilistic neural network |
| POL | Polynomial |
| PSO | particle swarm optimization |
| ROI | region of interest |
| SD | standard deviation |
| SPECT | single photon emission computed tomography |
| SVD | singular value decomposition |
| TJM | Trace of Jacobian matrix |
| US | undersampling |
| VFI | voting feature intervals |
| WTT | Welch’s t-test |
